# Metabolomics reveals altered metabolites in cirrhotic patients with severe portal hypertension in Tibetan population

**DOI:** 10.3389/fmed.2024.1404442

**Published:** 2024-06-28

**Authors:** Yanting Ye, Chao Xia, Hong Hu, Shihang Tang, Hui Huan

**Affiliations:** ^1^Lab of Gastroenterology and Hepatology, West China Hospital, Sichuan University, Chengdu, China; ^2^Department of Radiology, and Functional and Molecular Imaging Key Laboratory of Sichuan Province, West China Hospital, Sichuan University, Chengdu, China; ^3^Huaxi MR Research Center (HMRRC), West China Hospital, Sichuan University, Chengdu, China; ^4^Department of Gastroenterology, Hospital of Chengdu Office of People’s Government of Tibetan Autonomous Region, Chengdu, China; ^5^Department of Gastroenterology, Chongqing University Cancer Hospital, Chongqing, China

**Keywords:** Tibetan, liver cirrhosis, portal hypertension, metabolomics, biomarkers

## Abstract

**Background:**

Portal hypertension (PHT) presents a challenging issue of liver cirrhosis. This study aims to identify novel biomarkers for severe PHT (SPHT) and explore the pathophysiological mechanisms underlying PHT progression.

**Methods:**

Twenty-three Tibetan cirrhotic patients who underwent hepatic venous pressure gradient (HVPG) measurement were included. Eleven patients had an HVPG between 5 mmHg and 15 mmHg (MPHT), while 12 had an HVPG ≥16 mmHg (SPHT). Peripheral sera were analyzed using liquid chromatograph-mass spectrometer for metabolomic assessment. An additional 14 patients were recruited for validation of metabolites.

**Results:**

Seven hundred forty-five metabolites were detected and significant differences in metabolomics between MPHT and SPHT patients were observed. Employing a threshold of *p* < 0.05 and a variable importance in projection score >1, 153 differential metabolites were identified. A significant number of these metabolites were lipids and lipid-like molecules. Pisumionoside and N-decanoylglycine (N-DG) exhibited the highest area under the curve (AUC) values (0.947 and 0.9091, respectively). Additional differential metabolites with AUC >0.8 included 6-(4-ethyl-2-methoxyphenoxy)-3,4,5-trihydroxyoxane-2-carboxylic acid, sphinganine 1-phosphate, 4-hydroxytriazolam, 4,5-dihydroorotic acid, 6-hydroxy-1H-indole-3-acetamide, 7alpha-(thiomethyl)spironolactone, 6-deoxohomodolichosterone, glutaminylisoleucine, taurocholic acid 3-sulfate, and Phe Ser. Enzyme-linked immunosorbent assay further confirmed elevated levels of sphinganine 1-phosphate, N-DG, and serotonin in SPHT patients. Significant disruptions in linoleic acid, amino acid, sphingolipid metabolisms, and the citrate cycle were observed in SPHT patients.

**Conclusion:**

Pisumionoside and N-DG are identified as promising biomarkers for SPHT. The progression of PHT may be associated with disturbances in lipid, linoleic acid, and amino acid metabolisms, as well as alterations in the citrate cycle.

## Introduction

Liver cirrhosis presents the end stage of various kinds of chronic liver disease (CLD), characterized by the accumulation of extracellular matrix and distortion of hepatic vascular architecture (1). Globally, cirrhosis claims approximately one million lives annually (2). In China, it is estimated to affect 7 million (0.51%) of the population (3). Portal hypertension (PHT) is a major complication of cirrhosis (4). It is defined as increased pressure in the portal vein, typically identified by a hepatic venous pressure gradient (HVPG) exceeding 5 mmHg (5). The primary pathophysiological change in PHT is increased intrahepatic vascular resistance due to morphological changes within the liver. Factors such as reduced nitric oxide synthesis and increased portal blood flow further exacerbate PHT (6). Complications from PHT, including ascites, variceal bleeding, hepatic encephalopathy, and hepatorenal syndrome, are significant causes of emergencies, readmissions, and deaths among cirrhotic patients (5). Thus, PHT presents a troublesome problem in clinical management and is associated with a poor prognosis. Accurate assessment and early intervention for cirrhotic patients at high risk of severe PHT are of great importance to improve patient outcomes and reduce mortality and morbidity. Monitoring the pressure of the portal vein is also essential for assessing the effectiveness of pharmacological treatments and guiding therapeutic decisions (6).

Currently, HVPG remains the gold standard for diagnosing PHT (7). It is also invaluable for risk stratification, selecting appropriate treatments, evaluating PHT progression, and assessing treatment efficacy (8, 9). However, HVPG is acquired through catheterization of the hepatic veins (10). The invasive, demanding, and expensive features of HVPG limit its clinical application and hinder its widespread adoption in most medical centers. Non-invasive methods to measure PHT include Doppler ultrasound, liver transient elastography, magnetic resonance imaging, computed tomography, et al. However, inaccuracy in assessing the severity of PHT, limited universality, high cost, time-consuming, and radiation also restrict their extensive use (11, 12). Therefore, there is a significant need for developing a novel, non-invasive, economical, accurate, specific, and sensitive marker for the detection of PHT. This would facilitate regular monitoring of portal vein pressure and long-term management of PHT patients. Metabolomics is the study of metabolites with low molecular weight (e.g., <1.5 kDa) found in cells, biofluids, and tissues (13). The metabolome information can provide specific quantitative traits related to health and disease. The non-invasiveness and convenience of metabolomics make it widely employed in biomarker discovery and mechanisms elucidating (14).

The Tibetan population, residing in high-altitude regions, experiences extreme cold, low oxygen, and high ultraviolet exposure. This leads to unique physiological and medical traits valuable for medical research (15). However, medical research on the population in this region remains limited due to factors such as remote geography, harsh environments, and cultural differences. Metabolomic analysis of liver diseases is also a gap in understanding. The present study aimed to describe the metabolic profile of Tibetan cirrhotic patients with PHT and identify the unique metabolites and potential mechanisms associated with PHT progression (defined as an HVPG ≥16 mmHg).

## Materials and methods

### Ethical consideration

All subjects gave their informed consent for inclusion before they participated in the study. The study was conducted following the Declaration of Helsinki, and the protocol was approved by the Ethics Committee of the Hospital of Chengdu Office of People’s Government of Tibetan Autonomous Region (Research No. 64 of the Year 2020).

### Inclusion and exclusion criteria

Inclusion criteria: Cirrhotic patients hospitalized between November 2020 and June 2023 at the Hospital of the Chengdu Office of the People’s Government of the Tibetan Autonomous Region, which primarily provides medical services to the Tibetan population, were studied. Tibetan patients aged 18–75 years who met the diagnostic criteria for liver cirrhosis (16) and were going to undergo the HVPG measurement during their hospitalization were included. Tibetan ethnicity was confirmed by identification as Tibetan on a Resident Identity Card and long-term residency in the Tibetan Plateau.

Exclusion criteria: Pregnant or lactating women, patients with mental illnesses, or those with grade 2 or 3 hepatic encephalopathy were excluded.

A total of 23 patients were finally enrolled in the study. Based on HVPG, these patients were categorized into two groups: mild-to-moderate PHT (MPHT): 11 patients with an HPVG ranging from 5 mmHg to 15 mmHg; severe PHT (SPHT): 12 patients with an HPVG of 16 mmHg or higher. An additional cohort of 14 patients was included for the validation, adhering to the same inclusion and exclusion criteria. These patients were divided into two groups: validation-MPHT (VMPHT): 6 patients with an HPVG ranging from 5 mmHg to 15 mmHg; validation-SPHT (VSPHT): 8 patients with an HPVG of 16 mmHg or higher.

### Metabonomic analysis

Serum extraction: Peripheral blood samples were collected from the cirrhotic patients using serum-separator tubes without anticoagulants. The collected blood was allowed to clot at room temperature for 30 min to 1 h, avoiding any vibration or movement of the tubes. Following clotting, the samples were centrifuged at 4°C and 3,000 rpm for 15 min. The supernatant serum was carefully aspirated and aliquoted into sterile Eppendorf tubes. The serum samples were immediately stored at −80°C. Transportation was carried out on dry ice. All samples were analyzed under the same experimental conditions.

Metabonomic analysis: The serum underwent a liquid chromatograph-mass spectrometer (LC-MS) for further metabonomic analysis. In short, the samples were separated by liquid chromatography, and then the single component entered the ion source of the high vacuum mass spectrometer for ionization. The mass spectrum was obtained according to the separated mass-to-charge ratio (*m*/z). Denoising smoothing, baseline correction, and overlapping peak identification were applied to extract the information of metabolites. Finally, the qualitative and quantitative results of the samples were obtained by normalizing, data transforming, and standardizing the mass spectrum data.

### Validation of the differential metabolites

Serum levels of serotonin, sphinganine 1-phosphate (S1P), and N-decanoylglycine (N-DG) were measured using enzyme-linked immunosorbent assay (ELISA) kits from Jingmei Biotechnology. The assays were performed according to the manufacturer’s instructions. Optical density at 450 nm (OD_450_) was measured with a microplate reader, and the concentrations of the biomarkers were calculated using the standard curves provided with the kits.

### Data analysis

Continuous variables were presented as mean ± standard deviation (SD). For comparing means between two groups with equal variances, the student’s *t*-test was utilized. Conversely, when comparing means between two groups with unequal variances, Welch’s *t*-test was employed. A *p*-value of less than 0.05 was considered statistically significant. Principal component analysis (PCA) and partial least squares-discriminant analysis (PLS-DA) were used to overview the metabolic profile of the MPHT and SPHT groups. The PLS-DA models were evaluated based on R2X, R2Y, and Q2 as indicators of their quality. R2X and R2Y signify cumulative explanatory rates, with Q2 reflecting the model’s predictive capacity. Variable influence on projection (VIP) was obtained based on the orthogonal partial least square discriminant analysis (OPLS-DA) models. The differential metabolites were identified based on a combination of *p*-value and VIP. Metabolites were classified as differential if they met the criteria of *p* < 0.05 and VIP >1. Furthermore, metabolites that additionally exhibited a fold change (FC) greater than 1.2 under these conditions were categorized as significant differential metabolites. These analyses were conducted using the Majorbio Cloud Platform.^1^

## Results

### Characteristics of cirrhotic patients

A total of 23 cirrhotic patients who underwent HVPG were enrolled in the present study. Based on HVPG, the patients were divided into the MPHT group (*n* = 11) and the SPHT group (*n* = 12). The average HVPG of the MPHT group was 11.45 ± 2.07 mmHg, while it was 20.92 ± 3.8 mmHg in the SPHT group (*p* < 0.001). The main characteristics of the patients were summarized in Table 1. No significant differences were observed in the age, body mass index (BMI), and laboratory tests between the two groups.

**Table 1:** Clinical characteristics of cirrhotic patients.

	MPHT (*n* = 11)	SPHT (*n* = 12)
Average HVPG (mmHg)	11.45 ± 2.07	20.92 ± 3.80
Male (*n*, %)	6 (54.55%)	6 (50%)
BMI	27.04 ± 3.70	25.37 ± 3.77
Mean age (years)	54.78 ± 5.64	54.58 ± 9.25
Varices (*n*)	9 (*N* = 10)	10 (*N* = 11)
Variceal bleeding (*n*, %)	5 (45%)	5 (41.67%)
Ascites (*n*, %)	7 (63.64%)	9 (75%)
Hepatic encephalopathy (*n*, %)	0 (0)	1 (8.33%)
**Laboratory tests**		
ALT (IU/L)	52.18 ± 43.58	38.83 ± 31.92
AST (IU/L)	52.27 ± 46.93	48.00 ± 33.65
ALB (g/L)	33.90 ± 6.43	33.90 ± 7.60
Tbil (μmol/L)	28.08 ± 14.87	28.37 ± 22.12
Hb (g/L)	109.55 ± 20.42	118.50 ± 33.92
PLT (×10^9^/L)	71.27 ± 21.14	91.83 ± 32.65
Crea (μmol/L)	57.09 ± 7.53	84.25 ± 48.85
TC (mmol/L)	3.55 ± 0.92	2.95 ± 0.69
Triglycerides (mmol/L)	0.84 ± 0.22	0.73 ± 0.22
LDL (mmol/L)	2.14 ± 0.86	1.71 ± 0.56
FPG (mmol/L)	6.15 ± 3.04	5.11 ± 1.50
**Child-pugh (*n*)**		
A	2	4
B	7	6
C	2	2
**Etiology (*n*)**		
Hepatitis B	6	8
ALD	5	2
AIH	0	1
Cryptogenic	0	1

MPHT, mild-to-moderate portal hypertension; SPHT, severe portal hypertension; HVPG, hepatic venous pressure gradient; BMI, body mass index; ALT, alanine aminotransferase; AST, aspartate aminotransferase; ALB, albumin; Tbil, total bilirubin; Hb, hemoglobin; PLT, platelet; Crea, creatinine; TC, total cholesterol; LDL, low-density lipoprotein; FPG, fasting plasma glucose; ALD, alcoholic liver disease; AIH, autoimmune hepatitis.

### Metabolic profile of peripheral serum from cirrhotic patients

**Figure 1:**
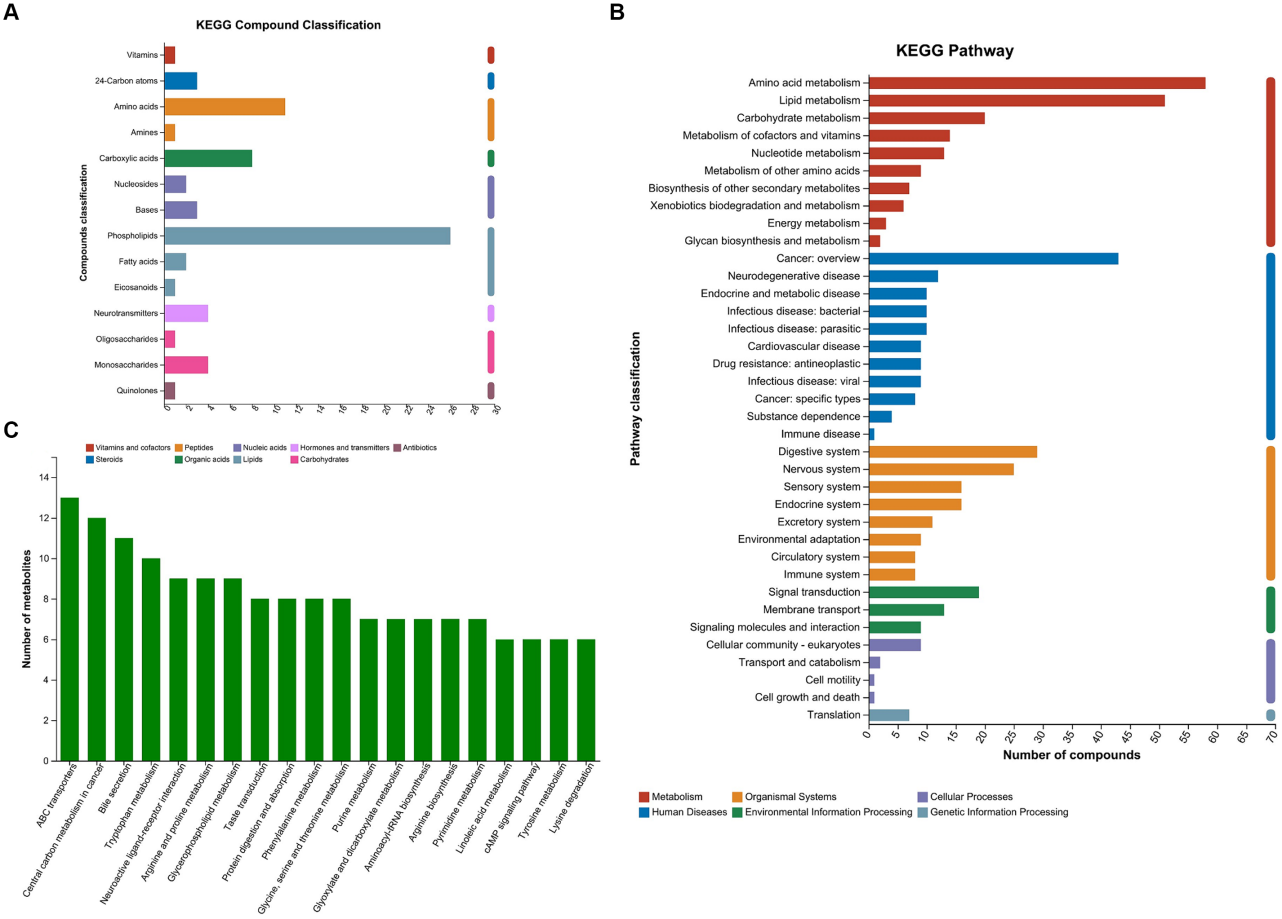
KEGG compound classification and KEGG pathways of the metabolites. **(A)** Metabolites were classified into 9 categories according to KEGG compound database. **(B)** The metabolites were annotated into KEGG pathways. **(C)** Top 20 KEGG pathways. KEGG, Kyoto Encyclopedia of Genes and Genomes; MPHT, mild-to-moderate portal hypertension; SPHT, severe portal hypertension.

A total of 745 metabolites (459 in positive ion mode and 286 in negative ion mode) were successfully identified. Among the metabolites, 593 were documented in the library. The termed metabolites were classified into 9 categories according to the Kyoto Encyclopedia of Genes and Genomes (KEGG) compound database. Phospholipids (26 metabolites), amino acids (11 metabolites), and carboxylic acids (8 metabolites) accounted for the most common categories (Figure 1A). The amino acid metabolism and lipid metabolism were the most frequent pathways that the metabolites were involved in Figure 1B. The top 20 KEGG pathways were shown in Figure 1C. The ATP-binding cassette (ABC) transporters, central carbon metabolism in cancer, bile secretion, and tryptophan metabolism presented the top four pathways.

**Figure 2:**
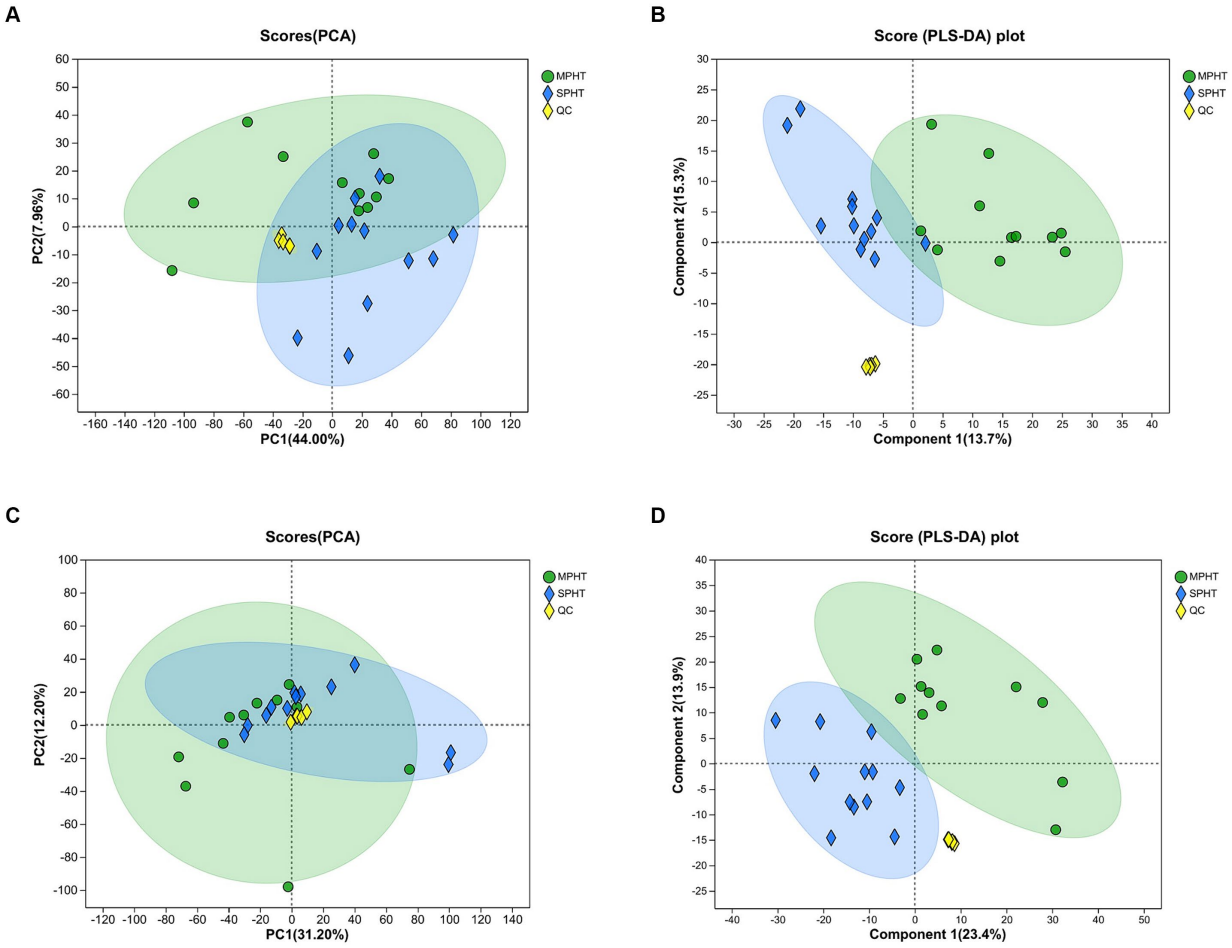
PCA and PLS-DA demonstrate the metabolic differences between the two groups. **(A,B)** PCA score plots between MPHT and SPHT groups in positive ion model **(A)** and negative ion model **(B)**. **(C,D)** PLS-DA score plots between MPHT and SPHT groups in positive ion model **(C)** and negative ion model **(D)**. PCA, principle component analysis; PLS-DA, partial least squares discriminant analysis; MPHT, mild-to-moderate portal hypertension; SPHT, severe portal hypertension.

PCA was applied to overview the metabolic differences between the MPHT and SPHT groups. A combination of overlapping and distinct components between the two groups was observed in PCA (Figures 2A,B). Considering this unsupervised analysis may miss the discrepancy between groups, PLS-DA was employed to identify the differences in metabolic profile. As shown in Figures 2C,D, the metabolic profiles of serum from the two groups in the positive and negative ion modes were separated, indicating the unique metabolic profile of severe PHT patients. The clustered quality control (QC) samples indicated the system’s stability and quality of the data. No outliers are present, and all values reside within the 95% confidence interval. Permutation testing showed the intercepts of Q2 = 0.831, R2X = 0.591, R2Y = 0.979 from the positive ion mode data and Q2 = 0.822, R2X = 0.536, R2Y = 0.98 from the negative ion mode data, indicating the model’s stability, reliability, and robust explanatory and predictive capabilities. Furthermore, the intercepts of the Q2 regression lines with the *Y*-axis, in both positive and negative modes, were below 0.05, affirming the absence of model overfitting (data not shown).

### Unique metabolites of SPHT

**Figure 3:**
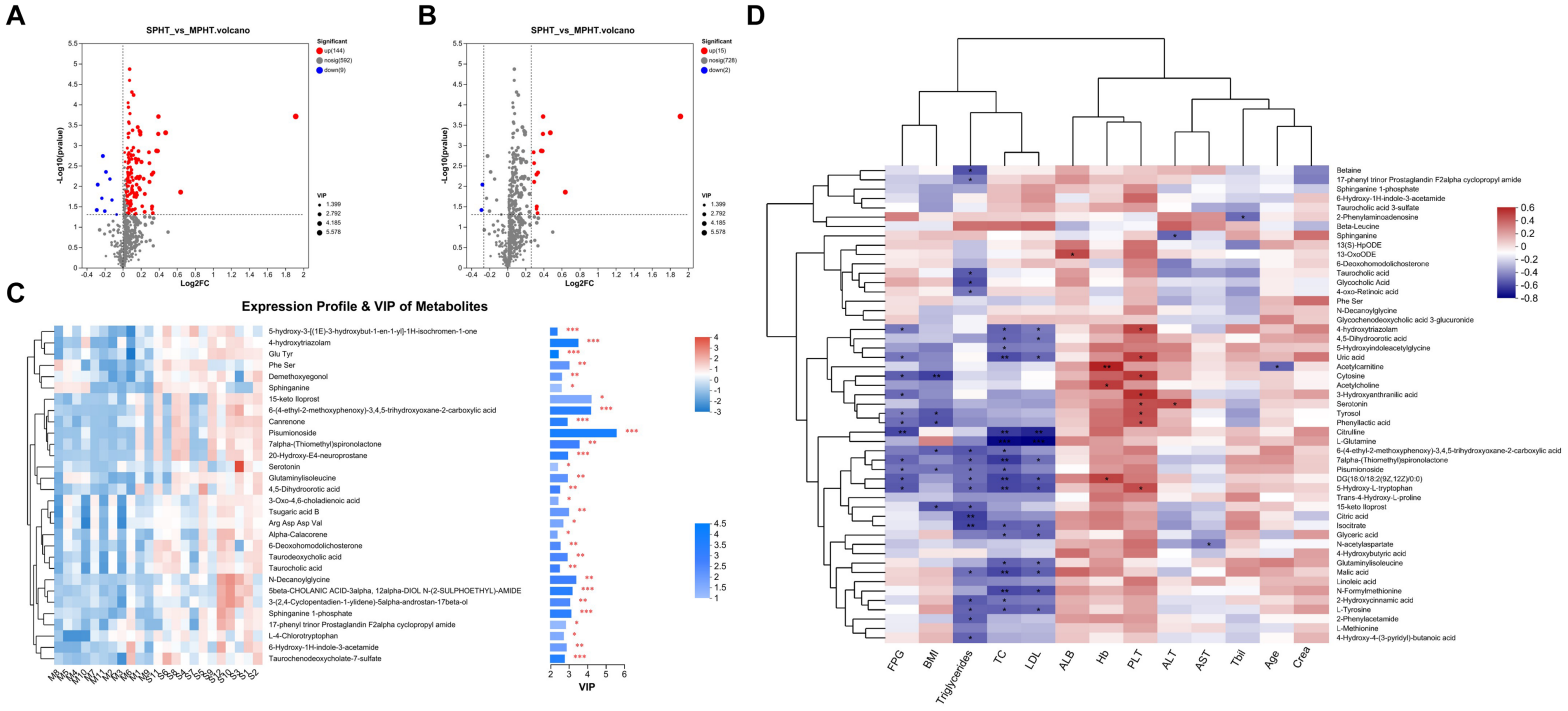
The differential metabolites between the MPHT and SPHT groups. **(A,B)** Volcano plots based on the OPLS-DA models. The differential metabolites were identified by the criteria of *p* < 0.05 and VIP >1 **(A)**, and *p* < 0.05, VIP >1 and FC >1.2 **(B)**. **(C)** Expression profile and VIP of the top 30 upregulated metabolites in SPHT patients. **(D)**The correlation heatmap of the clinical data and the differential metabolites. MPHT, mild-to-moderate portal hypertension; SPHT, severe portal hypertension; OPLS-DA, orthogonal partial least squares discriminant analysis; VIP, variable importance in projection; FC, fold change; FPG, fasting plasma glucose; BMI, body mass index; TC, total cholesterol; LDL, low-density lipoprotein; ALB, albumin; Hb, hemoglobin; PLT, platelet; ALT, alanine aminotransferase; AST, aspartate aminotransferase; Tbil, total bilirubin; Crea, creatinine.

The differential metabolites were selected based on criteria including *p* < 0.05 and VIP >1, leading to the identification of 153 differential metabolites (89 in positive ion mode and 64 in negative ion mode). The discrepancies in metabolite profiles between the two groups were illustrated in volcano plots (Figure 3A). Notably, most of these metabolites were significantly elevated in the SPHT group. Applying stricter criteria of *p* < 0.05, VIP >1, and FC >1.2, only 17 significant differential metabolites were identified, with 15 being significantly upregulated in the SPHT group (Figure 3B and Table 2). Annotation of these metabolites in the Human Metabolome Database (HMDB) revealed that they predominantly belong to the superclass of lipids and lipid-like molecules and the class of carboxylic acids and derivatives.

**Table 2:** The significant differential metabolites upregulated in the SPHT patients.

Metabolite	Library ID	HMDB Superclass	HMDB Class	VIP	FC	*p*-value
Pisumionoside	HMDB0039947	Lipids and lipid-like molecules	Prenol lipids	5.5777	3.7703	0.000196
15-keto iloprost	—	—	—	4.208	1.5583	0.01402
6-(4-ethyl-2-methoxyphenoxy)-3,4,5-trihydroxyoxane-2-carboxylic acid	—	—	—	4.191	1.389	0.000491
7alpha-(thiomethyl)spironolactone	—	—	—	3.5642	1.2948	0.001363
4-hydroxytriazolam	HMDB0061052	Organoheterocyclic compounds	Benzodiazepines	3.4919	1.3141	0.000197
N-decanoylglycine	HMDB0013267	Organic acids and derivatives	Carboxylic acids and derivatives	3.3855	1.3069	0.001375
Sphinganine 1-phosphate	HMDB0001383; HMDB0242462; 19,794–97-9	Lipids and lipid-like molecules	Sphingolipids	3.1129	1.3125	0.000525
Phe Ser	—	—	—	3.0076	1.2497	0.005162
Glutaminylisoleucine	HMDB0028800	Organic acids and derivatives	Carboxylic acids and derivatives	2.9307	1.2634	0.004651
6-hydroxy-1H-indole-3-acetamide	HMDB0031173	Organoheterocyclic compounds	Indoles and derivatives	2.8637	1.2271	0.007869
17-phenyl trinor prostaglandin F2alpha cyclopropyl amide	—	—	—	2.8281	1.2519	0.03163
6-deoxohomodolichosterone	HMDB0034430	Lipids and lipid-like molecules	Steroids and steroid derivatives	2.5361	1.2266	0.002732
4,5-dihydroorotic acid	HMDB0003349; HMDB0000528	Organic acids and derivatives	Carboxylic acids and derivatives	2.5085	1.222	0.001479
Serotonin	50–67-9; HMDB0000259	Organoheterocyclic compounds	Indoles and derivatives	2.3852	1.2589	0.04574
Taurocholic acid 3-sulfate	HMDB0002581; LMST05020031	Lipids and lipid-like molecules	Steroids and steroid derivatives	2.0362	1.2496	0.03614

SPHT, severe portal hypertension; VIP, variable importance in projection; FC, fold change (SPHT/MPHT).

The result of VIP analysis of the top 30 differential metabolites was shown in Figure 3C. Pisumionoside exhibited the most pronounced distinction between the two groups with the highest VIP and FC values (VIP = 5.58, FC = 3.77, *p* < 0.001). When annotated in the HMDB, a substantial proportion of the differential metabolites fell within the superclass of lipids and lipid-like molecules. This included pisumionoside, S1P, tsugaric acid B, 20-hydroxy-E4-neuroprostane, canrenone, taurodeoxycholic acid, taurochenodeoxycholate-7-sulfate, 6-deoxohomodolichosterone, taurocholic acid, 3-oxo-4,6-choladienoic acid, and alpha-calacorene. At the class level, N-DG, glutaminylisoleucine, and 4,5-dihydroorotic acid were categorized as carboxylic acids and derivatives. And 6-hydroxy-1H-indole-3-acetamide, L-4-chlorotryptophan, and serotonin were annotated to the class of indoles and derivatives.

Subsequent correlation analyses between primary clinical data and selected differential metabolites were performed (Figure 3D). Overall, blood lipids exhibited some correlation with the differential metabolites. Conversely, age, creatinine (Crea), total bilirubin (Tbil), aspartate aminotransferase (AST), albumin (ALB), and hemoglobin (Hb) did not show significant correlations. Specifically, pisumionoside demonstrated a negative correlation with body mass index (BMI), fasting plasma glucose (FPG), total cholesterol (TC), and triglycerides (TG). The metabolite 4-hydroxytriazolam showed a positive correlation with platelet count (PLT) and negative correlations with FPG, TC, and low-density lipoprotein (LDL). Metabolites related to the citrate cycle (TCA cycle) (citric acid, malic acid, and isocitrate) were found to be negatively correlated with blood lipids. Metabolites involved in tryptophan metabolism (serotonin, 5-hydroxyindoleacetylglycine, 3-hydroxyanthranilic acid, and 5-hydroxy-L-tryptophan) were correlated with alanine aminotransferase (ALT), PLT, blood lipids, and FPG, with serotonin showing positive correlations with ALT and PLT. N-decanoylglycine (N-DG), S1P, and linoleic acid metabolites (13(S)-HpODE, linoleic acid and 13-OxoODE) did not exhibit noticeable correlations with the clinical indicators.

### Enrichment and clustering of differential metabolites

When annotated to the KEGG compound library, a total of 35 differential metabolites were identified. Among these, the majority, totaling 18, were classified under compounds with biological roles, followed by 12 metabolites categorized as lipids. Phytochemical compounds and pesticides had 4 and 1 annotated metabolites, respectively. No metabolites were annotated to endocrine disrupting compounds and bioactive peptides. In terms of secondary classification, amino acids (compounds with biological roles: L-methionine, trans-4-hydroxy-L-proline, N-formylmethionine, L-tyrosine, citrulline, and L-glutamine), carboxylic acids (compounds with biological roles: citric acid, 4-hydroxybutyric acid, isocitrate and malic acid), and ST05 Steroid conjugates (lipids: taurodeoxycholic acid, glycocholic acid, taurocholic acid) had the highest number of annotated differential metabolites. The top 30 differential metabolites annotated to the KEGG compound library revealed that the majority of differential metabolites belong to lipids (5 metabolites).

**Figure 4:**
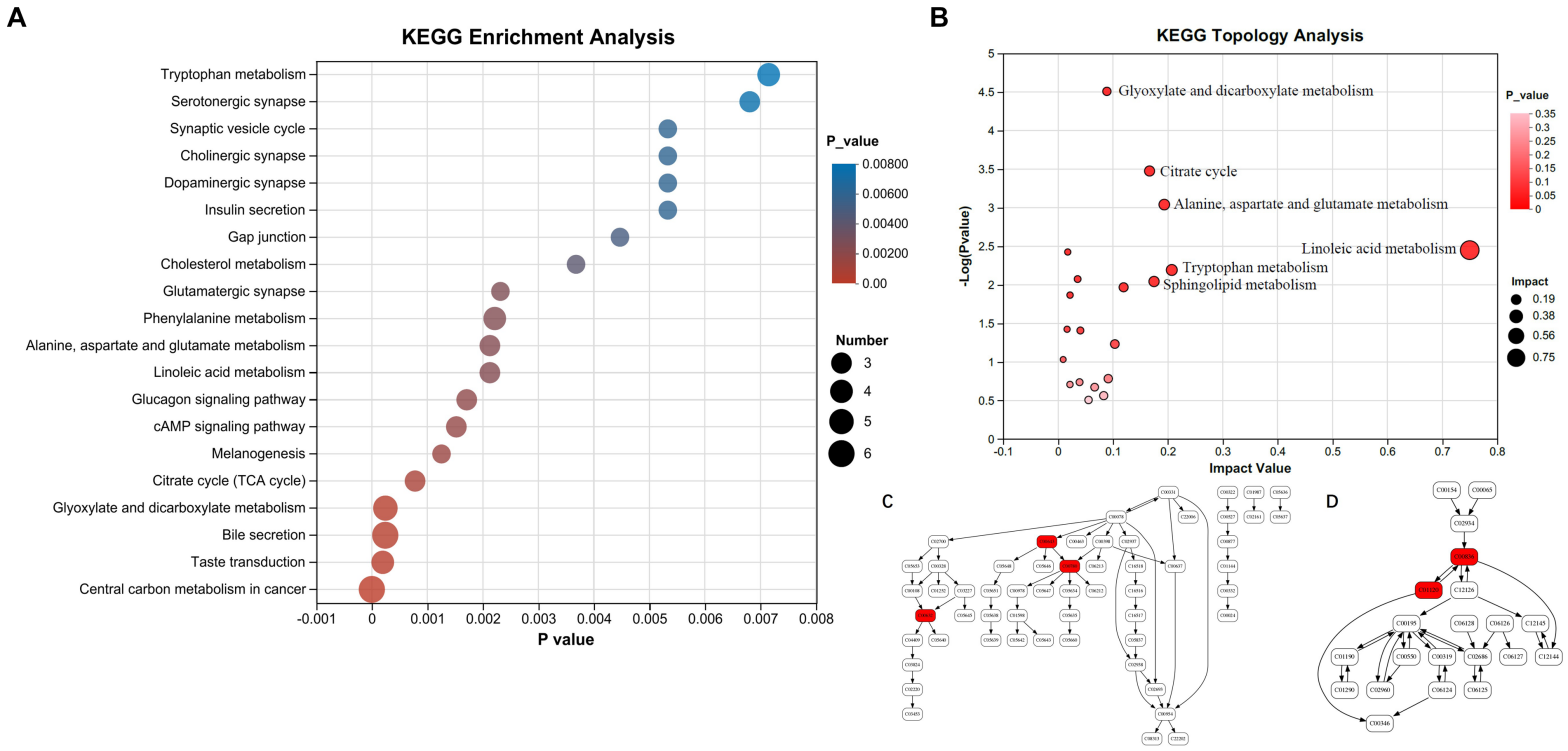
Enrichment analysis of differential metabolites in KEGG pathways. **(A)** KEGG pathway enrichment analysis. **(B)** Metabolic topology analysis. **(C)** Tryptophan metabolism pathway (serotonin, 5-hydroxy-L-tryptophan and 3-hydroxyanthranilic acid marked with red are significantly changed in peripheral blood plasma of SPHT patients). **(D)** Sphingolipid metabolism pathway (sphinganine and sphinganine 1-phosphate marked with red are significantly changed in peripheral blood plasma of SPHT patients). KEGG, Kyoto Encyclopedia of Genes and Genomes; MPHT, mild-to-moderate portal hypertension; SPHT, severe portal hypertension.

Upon alignment with the KEGG pathway database, the 17 differential metabolites exhibited enrichment in 20 pathways (Figure 4A). The central carbon metabolism (CCM) pathway exhibited the most significant enrichment of differential metabolites, and it contains the highest number of such metabolites (6 metabolites). CCM includes glycolysis, the TCA cycle, and the pentose phosphate pathway. It is the most fundamental metabolic process, providing energy and metabolic precursors for the body (17). The enrichment of differential metabolites in the bile secretion pathway was also significant (6 metabolites). Additionally, the glyoxylate and dicarboxylate metabolism, taste transduction, phenylalanine metabolism, and tryptophan metabolism pathways all presented significant enrichment of differential metabolites. The KEGG enrichment analysis of the top 30 differential metabolites revealed that the pathways most significantly involved were the sphingolipid metabolism and the neuroactive ligand-receptor interaction pathway, where serotonin and S1P participated (data not shown).

KEGG pathway enrichment analysis showed that the differential metabolites were significantly enriched in the following pathways: Glyoxylate and dicarboxylate metabolism (impact factor 0.00), TCA cycle (impact factor 0.17), alanine, aspartate, and glutamate metabolism (impact factor 0.19), linoleic acid metabolism (impact factor 0.75), tryptophan metabolism (impact factor 0.21), and sphingolipid metabolism (impact factor 0.17) (Figure 4B). The impact factor denotes the relative importance of metabolites within each pathway. Network diagrams presented in Figure 4C and Figure 4D illustrated the positions of differential metabolites within the tryptophan and sphingolipid metabolism pathways. In Figure 4C, the compound C00630 refers to 5-Hydroxy-L-tryptophan, C00780 is identified as serotonin, and C00632 corresponds to 3-Hydroxyanthranilic acid. In Figure 4D, C00836 is sphinganine and C01120 represents S1P.

### Identification of the potential biomarkers for SPHT

**Figure 5:**
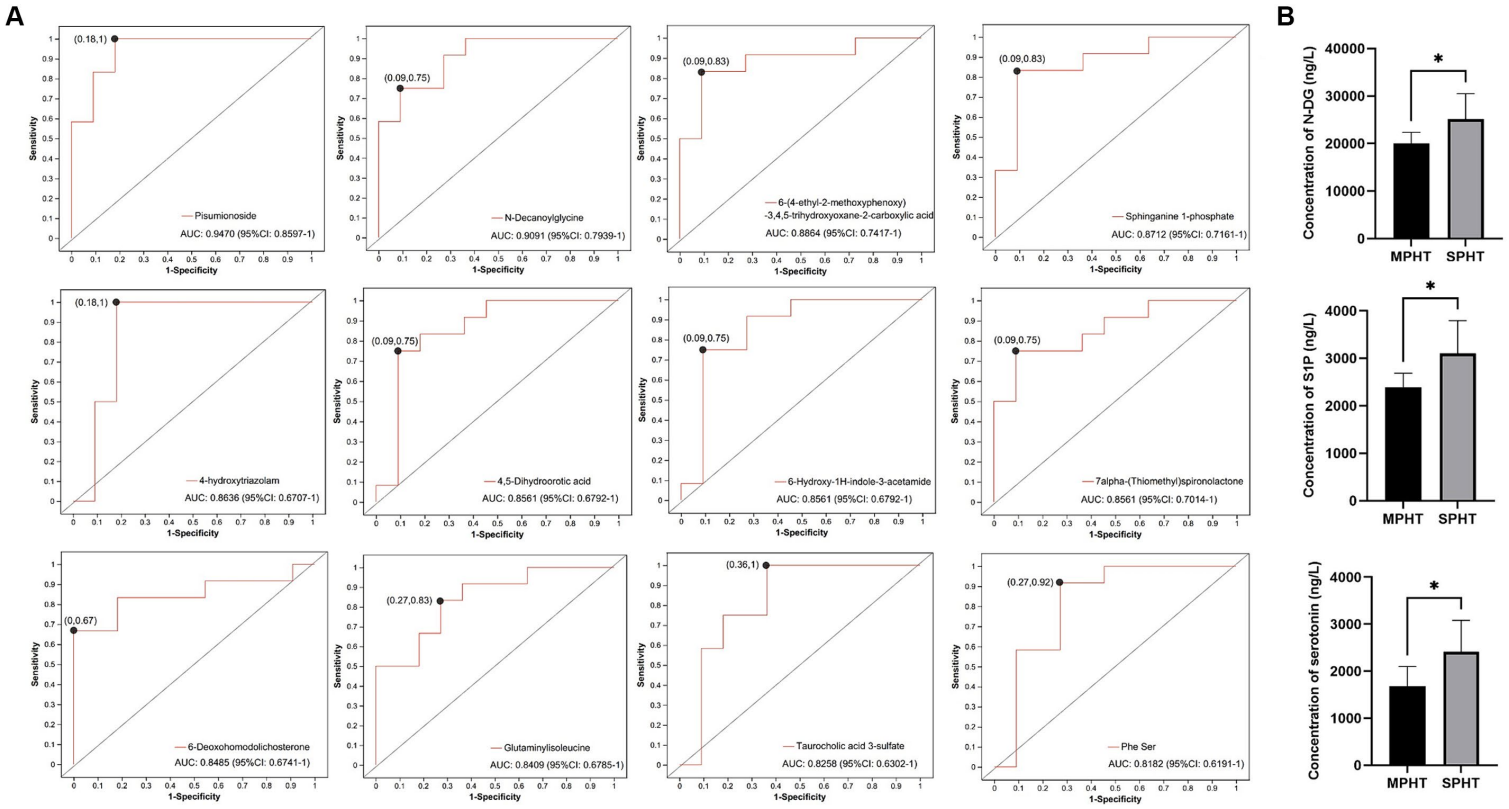
ROC curve analysis and ELISA validation of the significant differential metabolites. **(A)** ROC curves of the significant differential metabolites which have an AUC >0.8. **(B)** The serum concentrations of N-DG, S1P and serotonin using ELISA kits. ^*^p < 0.05. ROC, receiver operating characteristic; AUC, area under the curve; ELISA, enzyme-linked immunosorbent assay; N-DG, N-decanoylglycine; S1P, sphinganine 1-phosphate.

To assess the diagnostic capability of differential metabolites for SPHT, we conducted a receiver operating characteristic (ROC) curve analysis on the 15 significantly elevated differential metabolites in SPHT patients. The following differential metabolites presented area under the curve (AUC) >0.8 through ROC analysis: pisumionoside (0.947), N-DG (0.9091), 6-(4-ethyl-2-methoxyphenoxy)-3,4,5-trihydroxyoxane-2-carboxylic acid (0.8864), S1P (0.8712), 4-hydroxytriazolam (0.8636), 4,5-dihydroorotic acid (0.8561), 6-hydroxy-1H-indole-3-acetamide (0.8561), 7alpha-(thiomethyl)spironolactone (0.8561), 6-deoxohomodolichosterone (0.8485), glutaminylisoleucine (0.8409), taurocholic acid 3-sulfate (0.8258) and Phe Ser (0.8182) (Figure 5A).

As two differential metabolites with an AUC greater than 0.9, pisumionoside and N-DG also exhibited high VIP values and significant *p*-values, underscoring their potential as biomarkers for SPHT. Additionally, S1P demonstrated not only a significant difference in levels between the two groups but also its involvement in sphingolipid metabolism and the neuroactive ligand-receptor interaction pathways. Sphingolipid metabolism is crucial for the integrity of cell membranes, apoptosis, proliferation, and aging. The catabolism of sphingolipids generates ceramide and sphingosine, which are ultimately degraded into S1P (18). Then S1P works as a signaling lipid that regulates many cellular processes (19). The neuroactive ligand-receptor interaction pathway encompasses a group of receptors and ligands located on the plasma membrane and involves in various signaling processes (20). These suggested the significant role of S1P in the progression of PHT. To further validate whether these significant different metabolites were elevated in SPHT patients, we conducted ELISA on the relevant metabolites in the validation group. The results showed that N-DG and S1P were significantly higher in VSPHT patients compared to VMPHT patients (*p* < 0.05) (Figure 5B). The clinical information of the validation patients was listed in Supplementary Tables S2, S3. Except for HVPG, there were no significant differences in age, BMI, or laboratory test results between the two groups.

Although the AUC value of serotonin was less than 0.8, its involvement in various vital physiological processes, such as bile secretion, tryptophan metabolism, neuroactive ligand-receptor interaction, and taste transduction, suggested a close association with the development and progression of PHT. Therefore, we also verified its levels using ELISA. The result showed that serum serotonin in VSPHT patients was significantly higher than in VMPHT patients (*p* < 0.05), consistent with our metabolomic analysis.

## Discussion

PHT represents a significant pathological alteration associated with cirrhosis, closely linked to severe complications and a poor prognosis in affected individuals. It has been documented that an HVPG exceeding 16 mmHg independently predicts mortality in cirrhotic patients, with values of 20 mmHg or higher indicating an extremely high risk of death (21). Therefore, measuring PHT is critical for identifying patients at high risk of complications and mortality, as well as for assessing responses to pharmacological treatment. Although HVPG is a highly accurate method for evaluating PHT, its invasive nature and technical demands limit its widespread application. Other non-invasive diagnostic methods for PHT include liver elastography, spleen elastography, and serum biomarkers (22). The high demands on equipment and operators for liver and spleen elastography, along with its accuracy being easily affected by obesity, limit its widespread application (23). Serum biomarkers offer high applicability, good reproducibility, and strong availability, making them highly promising for the non-invasive detection of PHT. Currently, available serum biomarkers include PLT count, Von Willebrand factor antigen, AST to PLT ratio index, AST-to-ALT ratio, Fibrosis-4 index, FibroIndex, and King’s score, et al. (22). However, these biomarkers can be influenced by comorbidities or medications. Furthermore, many of these biomarkers are primarily used to assess liver fibrosis, and their accuracy in diagnosing PHT is limited, particularly when used alone. Though other biomarkers such as soluble cluster of differentiation 163 (CD163) (24), inflammatory markers (25), and serum bile acids (26) have been investigated, current evidence is insufficient to recommend their routine use in clinical practice. Moreover, most existing biomarkers are primarily used to identify PHT with portal pressure greater than 10 mmHg or 12 mmHg (22, 27–29), lacking the ability to accurately identify more severe cases.

Recently, metabolomics sprung up to be a prospective way of discovering noninvasive biomarkers. Metabolites, as the end products of cellular processes, reflect the change in body condition and hint at the molecular mechanism in the development of diseases. The identification of unique and significantly altered metabolites in patients with SPHT could serve as non-invasive biomarkers for assessing portal pressure. The present study focused on the serum metabolomic alterations in cirrhotic patients with particularly severe PHT (PHT ≥16 mmHg) and identified several new potential biomarkers, providing additional options for the combined application of serum biomarkers in assessing portal pressure. The potential mechanisms driving the progression of PHT through metabolomic analysis were also explored.

Our study is particularly significant as it focuses on the Tibetan population, a group residing in high-altitude areas characterized by chronic hypoxia. This condition not only affects the hematopoietic system but also has implications for other organs. The liver, serving as a central hub for numerous metabolic processes and having a high oxygen demand, is susceptible to structural and functional changes under hypoxic conditions (30). Also, hypoxia-induced inflammation and disruptions in other organs may exert an influence on liver function (30, 31). Previous studies have suggested that hypoxia affects the lipid metabolism of the liver (32). Given these factors, it is plausible that the Tibetan population exhibits unique metabolomic profiles. Nevertheless, there is currently limited research on liver metabolomics among Tibetan populations. Our study addresses this gap by documenting the serum metabolomic profiles of Tibetan patients with liver cirrhosis.

Our study revealed that the majority of the top 30 differential metabolites are lipids and lipid-like molecules, and KEGG pathway analysis also indicated that these differential metabolites are predominantly enriched in pathways related to lipid metabolism. This suggests notable changes in lipid metabolism in SPHT patients compared to MPHT patients. The liver plays a pivotal role in the synthesis, storage, and metabolic processing of lipids and lipoproteins. The previous animal studies indicated that prehepatic PHT impaired liver lipid metabolism, primarily presented as increased lipid deposition and reduced phospholipid synthesis (33, 34). Furthermore, steatosis progressed with the advancement of PHT (33). Neuroendocrine disorder and immunologic factors resulting from cirrhotic PHT may contribute to the altered lipid metabolism. The potential neuroendocrine factors primarily include elevated levels of plasma corticoids, catecholamines, and glucagon. As for the immunologic factors, tumor necrosis factor α (TNF-α) is suggested to exert the most significant impact (33). It can influence lipid metabolism by promoting lipogenesis, inducing lipolysis, inhibiting the activity of lipid metabolism-related enzymes, and regulating cholesterol metabolism and adipokines derived from other adipocytes (35). However, the causal relationship between lipid metabolism disorder and the progression of PHT remains unclear, and they may mutually influence each other causally. Differential metabolites may play a crucial role in this interaction.

S1P is one of the significant differential metabolites. It is a bioactive lipid signaling molecule that participates in regulating inflammation, angiogenesis, vascular permeability, liver regeneration, cancer growth, and metastasis, et al. (19, 36). Inflammation was suspected to be a key mediator of the pathogenesis and severity of PHT (37). Angiogenesis and decreased sinusoidal permeability also play an important role in the pathogenesis of PHT (38). What’s more, S1P is a pivotal regulator in sphingolipid metabolism, capable of influencing the progression of liver fibrosis by modulating the hepatic immune response (39). It is reasonable to speculate that PHT-induced alterations in lipid metabolism upregulate S1P, which in turn promotes the progression of PHT. Despite the need for further research to confirm the role of S1P in the progression of PHT, it is still considered a promising biomarker for SPHT, supported by its significant elevation in PHT patients and its high AUC value.

Pisumionoside is a noteworthy differential metabolite categorized under lipids and lipid-like molecules. It is a membrane stabilizer and participates in biological processes including lipid peroxidation, fatty acid metabolism, cell signaling, and lipid metabolism (40). This is consistent with our findings: serum levels of pisumionoside were negatively correlated with blood lipids and glucose levels. These results suggest that elevated pisumionoside is associated with the dysregulated carbohydrate and lipid metabolism resulting from cirrhosis progression. Further research is needed to elucidate how these pathways influence pisumionoside. Although pisumionoside is associated with carbohydrate and lipid metabolism, our study did not observe an enrichment of pisumionoside in these metabolic pathways. Therefore, we tend to consider it as a consequence of PHT progression. Additionally, given its high AUC value, we believe it holds promise as a potential biomarker for SPHT.

N-DG is another differential metabolite with a particularly high AUC, suggesting its potential as a biomarker for SPHT as well. It is an acylglycine with a C-10 fatty acid group as the acyl moiety. It is reported to be elevated in patients with fatty acid oxidation disorders (41). However, our correlation analysis showed that N-DG is not significantly associated with blood lipids. Further research is necessary to explore the reasons behind the elevated N-DG level and its association with lipids metabolism.

KEGG pathway enrichment analysis revealed notable enrichment in the tryptophan metabolism pathway. Both 5-hydroxy-L-tryptophan and serotonin are positioned prominently within this pathway. 5-hydroxy-L-tryptophan, which is both a drug and a natural component of some dietary supplements, can degrade to serotonin (42). Serotonin is involved in various metabolic pathways, such as neuroactive ligand-receptor interaction, bile secretion, cAMP signaling pathway, gap junction, inflammatory mediator regulation of TRP channels, and synaptic vesicle cycle, suggesting a potential close association with the progression of PHT. Studies have reported that serotonin can modulate portal and sinusoidal blood flows (43). Injecting serotonin into the portal vein markedly increases portal vein pressure (44). Given the significant role of serotonin in the progression of PHT, elucidating the mechanisms underlying elevated serotonin levels in cirrhosis may aid in controlling the advancement of PHT. On one hand, elevated serotonin may be associated with hepatocyte damage during liver cirrhosis. Our correlation analysis showed that serotonin levels are positively correlated with serum ALT levels, which are released from damaged hepatocytes. Studies indicate that serotonin plays a crucial role in liver regeneration. Serotonin derived from PLT promotes liver regeneration following partial hepatectomy (45). Therefore, increased serotonin levels may be a result of the repair response to hepatocyte damage. However, elevated serotonin levels may promote the development of liver cirrhosis through various mechanisms we previously mentioned. On the other hand, in the periphery, most of the serotonin is synthesized by enterochromaffin cells located in the gut (46). It is widely acknowledged that microbial products play a significant role in influencing gut function (47). The synthesis and release of serotonin by enterochromaffin cells in the gut are influenced by intestinal microbiota through the production of short-chain fatty acids. Simply put, acetate and butyrate, produced by gut microbes from the fermentation of dietary sugars, boost the expression of TpH1 mRNA and raise serotonin production by enterochromaffin cells (48). The gut microbiota of patients with cirrhosis undergoes alterations, with distinct differences between compensated and decompensated stages (49). The gut microbiota of patients with portal hypertension was also different from other populations (50). However, the exact link between PHT and gut microbiota remains elusive. Additionally, differences in gut microbiota among patients with PHT at different stages have not been reported. Our study suggests that serotonin may serve as a key molecule bridging the gap between gut microbiota and the progression of PHT. Further research is required to elucidate which microbiota and their metabolites play pivotal roles in the progression of PHT.

Linoleic acid metabolism was remarkably disturbed in SPHT. The significantly elevated linoleic acid metabolites (13(S)-HpODE, 13-OxoODE, and linoleic acid) in SPHT patients in the present study hint at their role in the progression of PHT. Changes in linoleic acid metabolism have been reported in cirrhosis patients previously. Some studies found a decreased linoleic acid level in cirrhotic patients (51, 52). While EKODE, the metabolites from linoleic acid metabolism, was reported to be increased in patients with acutely decompensated cirrhosis and acute-on-chronic liver failure and be associated with a 28-day mortality of cirrhosis (53). In total, linoleic acid metabolism is suggested to play a critical role in influencing the incidence and progression of liver cirrhosis.

Our research also confirms that the TCA cycle is significantly disordered in patients with SPHT. Perturbations of TCA cycle metabolism have been reported to occur from the onset of liver fibrosis in carbon tetrachloride-induced fibrosis animal models (54). The TCA cycle is the ultimate common oxidative pathway for lipids, carbohydrates, and amino acids and it is the most important pathway connecting almost all metabolic pathways (55). Existing research has shown that the TCA cycle promotes insulin resistance and fatty liver by inducing mitochondrial dysfunction (56). This is consistent with the result of our correlation analysis, where metabolites of the TCA cycle were positively correlated with blood lipid levels. The inflammatory environment in cirrhotic PHT may lead to remodeling of the TCA cycle, resulting in alterations in the pro-inflammatory and anti-inflammatory metabolites (57). Our study uncovered a notable increase in citrate, one of the metabolites of the TCA cycle, in the peripheral serum of cirrhotic patients with SPHT. Citrate plays a crucial role in bridging carbohydrate and fatty acid metabolism, as well as in protein modification. Moreover, citrate functions as a significant inflammatory signal, modulating dendritic cell activation and the production of pro-inflammatory factors (58). Previous studies have shown a positive correlation between circulating citrate levels and the degree of liver fibrosis, with citrate levels increasing as fibrosis worsens (59). However, there is a lack of relevant research on citrate in PHT. Our study suggests that citrate may play a crucial role in the progression of PHT. Nevertheless, the elevation of citrate is a protective feedback response to inflammation and injury or a factor promoting PHT requires further investigation.

Although our study has identified potential biomarkers for SPHT and molecules that may be associated with the progression of PHT, there are some limitations to our research. A primary limitation is the small sample size. Expanding the sample size would be critical for further verifying the roles of these metabolites. Also, the diagnostic specificity and sensitivity of these biomarkers, in comparison to existing ones, need further investigation. Additionally, the roles of the metabolites in the progression of PHT require further investigation to be clearly defined.

## Conclusion

Tibetan cirrhotic patients who suffer from SPHT exhibit a distinct metabolomic profile compared to those with MPHT. Key metabolites such as pisumionoside and N-DG demonstrate high AUC values, showcasing their significant potential as biomarkers for SPHT. Additional metabolites including 6-(4-ethyl-2-methoxyphenoxy)-3,4,5-trihydroxyoxane-2-carboxylic acid, S1P, 4-hydroxytriazolam, 4,5-Dihydroorotic acid, 6-Hydroxy-1H-indole-3-acetamide, 7alpha-(Thiomethyl)spironolactone, 6-Deoxohomodolichosterone, Glutaminylisoleucine, Taurocholic acid 3-sulfate, and Phe Ser also emerge as promising biomarkers for SPHT. Notably, certain lipid metabolites, particularly S1P, are significantly elevated in SPHT patients, indicating that disruptions in lipid metabolism may be a key factor in the progression of PHT. Furthermore, metabolic pathways such as tryptophan metabolism, linoleic acid metabolism, and the citrate cycle are intricately linked with the pathophysiology of liver cirrhosis and PHT. These pathways may play pivotal roles in the progression of PHT. Further studies are essential to corroborate the influence of these metabolic pathways on PHT development and to decipher their specific molecular mechanisms.

## Data availability statement

The datasets presented in this study can be found in online repositories. The names of the repository/repositories and accession number(s) can be found in the article/Supplementary material.

## Ethics statement

The studies involving humans were approved by Ethics Committee of Hospital of Chengdu Office of People’s Government of Tibetan Autonomous Region (Research No. 64 of the Year 2020). The studies were conducted in accordance with the local legislation and institutional requirements. The participants provided their written informed consent to participate in this study.

## Author contributions

YY: Conceptualization, Visualization, Methodology, Investigation, Formal analysis, Data curation, Writing – original draft. CX: Visualization, Investigation, Funding acquisition, Writing – original draft, Methodology, Formal analysis. HoH: Writing – original draft, Visualization, Software, Methodology. ST: Writing – original draft, Visualization, Software, Methodology, Funding acquisition. HuH: Writing – review & editing, Validation, Supervision, Resources, Project administration, Funding acquisition, Conceptualization.

## Footnotes

^1^ www.majorbio.com
