# Supplementary material for: Metabolomics reveals altered metabolites in cirrhotic patients with severe portal hypertension in Tibetan population
Source: Front Med (Lausanne). 2024 Jun 28;11:1404442. doi: 10.3389/fmed.2024.1404442 (PMC11250582; doi:10.3389/fmed.2024.1404442)
Supplement: Supplementary file 1 [file Table_1.DOCX]

**Supplementary Table 1 Comorbidities and medications of cirrhotic patients**

| **Patient ID** | **Comorbidities** | **Medications** |
| --- | --- | --- |
| **M1** | Early-stage esophageal carcinoma; hypertension; diabetes mellitus; Helicobacter pylori infection; reflux esophagitis; duodenal bulbitis; gallstones. | **-** |
| **M2** | HCC; pulmonary infection; diabetes mellitus; gallstones; umbilical hernia. | Insulin aspart; furosemide; spironolactone. |
| **M3** | HCC; iron-deficiency anemia; Helicobacter pylori infection; hypokalemia. | **-** |
| **M4** | Diabetes mellitus; first-degree atrioventricular block; intestinal dysbiosis. | **-** |
| **M5** | Gallstones; internal hemorrhoids; hypokalemia. | **-** |
| **M6** | HCC; diabetes mellitus; hyperuricemia; liver cyst; Helicobacter pylori infection. | **-** |
| **M7** | Thyroid nodule. | Entecavir; tenofovir; sanguisorba officinalis. |
| **M8** | Duodenal bulb polyp; Suspicious right upper lung mass. | - |
| **M9** | Hypoproteinemia; chronic non-atrophic gastritis. | Tenofovir. |
| **M10** | - | - |
| **M11** | HCC; hepatic artery-portal vein fistula; hypertension; diabetes mellitus; hypokalemia. | Metformin. |
| **S1** | Chronic non-atrophic gastritis; osteoporosis; anemia. | - |
| **S2** | Gallstones. | - |
| **S3** | Hypoproteinemia; diabetes mellitus; diabetic peripheral neuropathy; chronic kidney disease; renal anemia. | Shenshuaining Capsule (pseudostellaria root; coptis root; prepared pinellia; tangerine peel; poria; rhubarb; salvia root; achyranthes root; safflower; licoric); α-Keto acid; Entecavir; furosemidel; spironolactone. |
| **S4** | Leukopenia. | **-** |
| **S5** | Gastroesophageal reflux disease. | **-** |
| **S6** | Hypertension; mild multiple splenic nodules; anemia; mild mitral and tricuspid valve regurgitation. | **-** |
| **S7** | Hypertension; diabetes mellitus. | **-** |
| **S8** | Moderate anemia; renal cysts. | **-** |
| **S9** | Low-grade intraepithelial neoplasia of the gastric antrum; chronic atrophic gastritis; hypertension; carotid atherosclerosis; severe anemia; gallstones; duodenal bulb polyp. | **-** |
| **S10** | Duodenal bulb ulcer; chronic non-atrophic gastritis; moderate anemia; hypocalcemia; hypophosphatemia; renal insufficiency. | **-** |
| **S11** | - | **-** |
| **S12** | Pulmonary infection. | **-** |

**Supplementary Table 2 Clinical characteristics of the validation patients**

|  | **VMPHT (n=6)** | **VSPHT (n=8)** |
| --- | --- | --- |
| **Average HVPG (mmHg)** | 12.33 ± 3.39 | 18.13 ± 2.30 |
| **Male (n, %)** | 5 (83.33%) | 6 (75%) |
| **BMI** | 25.79 ± 4.60 | 23.59 ± 3.86 |
| **Mean age (years)** | 50.50 ± 12.73 | 57.4 ± 8.76 |
| **Laboratory tests** |  |  |
| **ALT (IU/L)** | 21.67 ± 8.07 | 33.00 ± 32.76 |
| **AST (IU/L)** | 25.67 ± 6.98 | 44.25 ± 32.53 |
| **ALB (g/L)** | 32.20 ± 2.92 | 31.31 ± 5.10 |
| **Tbil (μmol/L)** | 25.93 ± 6.77 | 29.00 ± 18.00 |
| **Hb (g/L)** | 101.50 ± 38.87 | 112.75 ± 36.71 |
| **PLT (×10⁹/L)** | 89.00 ± 42.86 | 103.38 ± 59.65 |
| **Crea (μmol/L)** | 64.17 ± 18.21 | 58.25 ± 22.04 |
| **TC (mmol/L)** | 3.00 ± 0.63 | 3.32 ± 0.49 |
| **Triglycerides (mmol/L)** | 0.66 ± 0.12 | 0.73 ± 0.19 |
| **LDL (mmol/L)** | 1.61 ± 0.28 | 1.50 ± 0.38 |
| **FPG (mmol/L)** | 7.41 ± 3.52 | 6.25 ± 2.02 |
| **Child-Pugh (n)** |  |  |
| **A** | 3 | 2 |
| **B** | 3 | 5 |
| **C** | 0 | 1 |
| **Etiology (n)** |  |  |
| **Hepatitis B** | 1 | 2 |
| **ALD** | 3 | 4 |
| **Hepatitis B + ALD** | 1 | 1 |
| **Cryptogenic** | 1 | 1 |

VMPHT, validation-mild-to-moderate portal hypertension; VSPHT, validation-severe portal hypertension; HVPG, hepatic venous pressure gradient; BMI, body mass index; ALT, alanine aminotransferase; AST, aspartate aminotransferase; ALB, albumin; Tbil, total bilirubin; Hb, hemoglobin; PLT, platelet; Crea, creatinine; TC, total cholesterol; LDL, low-density lipoprotein; FPG, fasting plasma glucose; ALD, alcoholic liver disease.

.

**Supplementary Table 3 Comorbidities and medications of the validation patients**

| **Patient ID** | **Comorbidities** | **Medications** |
| --- | --- | --- |
| **M12** | Diabetes mellitus; gallstones; dysbiosis; hypokalemia; multiple colonic polyps. | **-** |
| **M13** | Deep vein thrombosis; thrombophilia. | Spironolactone; folic acid; polysaccharide iron. |
| **M14** | Chronic non-atrophic gastritis; gallstones; avascular necrosis of the right femoral head with severe osteoarthritis; subluxation of the right hip joint; degenerative changes of the sacroiliac joint; Helicobacter pylori infection; anemia; hypokalemia. | **-** |
| **M15** | Diabetes mellitus; hypokalemia. | Insulin; metformin. |
| **M16** | Hepatic mass; diabetes mellitus; hypertension; anemia. | Metformin. |
| **M17** | Anemia; fatty liver; Helicobacter pylori infection; renal cyst; prostatic hyperplasia; emphysema; hypokalemia. | Entecavir. |
| **S13** | Hypertension; hypokalemia. | Nifedipine. |
| **S14** | Cholestasis; emphysema. | - |
| **S15** | Gastric ulcers; chronic atrophic gastritis; hyperuricemia; anemia; gallstones; hiatal hernia. | **-** |
| **S16** | Anemia; dysbiosis; eczema. | **-** |
| **S17** | Electrolyte imbalance. | **-** |
| **S18** | Anemia; nephrolithiasis. | Entecavir. |
| **S19** | Gastric ulcer; pyloric stenosis; gastric retention; dysbiosis. | Entecavir. |
| **S20** | Dysbiosis; venous sclerosis; post-hepatectomy (after liver cancer surgery). | **-** |
